# Effects of smartphone addiction on cognitive function and physical activity in middle-school children: a cross-sectional study

**DOI:** 10.3389/fpsyg.2023.1182749

**Published:** 2023-08-14

**Authors:** Ahlam Al-Amri, Sahar Abdulaziz, Shahid Bashir, Mohammad Ahsan, Turki Abualait

**Affiliations:** ^1^Department of Physical Therapy, College of Applied Medical Sciences, Imam Abdulrahman Bin Faisal University, Dammam, Saudi Arabia; ^2^Rehabilitation Health Services, Armed Forces Hospital Southern Region, Abha, Saudi Arabia; ^3^Neuroscience Center, King Fahad Specialist Hospital, Dammam, Saudi Arabia

**Keywords:** smartphone addiction, children, cognitive function, physical activity, memory, attention

## Abstract

**Introduction:**

This study aimed to investigate the effects of smartphone addiction on cognitive function and physical activity in middle-school children.

**Methods:**

A population of 196 children (boys and girls) from middle schools were recruited for this study with an average age of 12.99 ± 0.81 years, a height of 153.86 ± 6.50 meters, a weight of 48.07 ± 7.31 kilograms, and a body mass index of 20.22 ± 2.08 kg/m^2^. Smartphone addiction was determined using Arabic versions of the Smartphone Addiction Scale-Short Version, and physical activity levels were assessed by a physical activity questionnaire for older children. The working memory and selective attention domains of cognitive function were evaluated using a laptop screen's digital version of the memory automaticity and Flanker tasks, respectively. A one-way MANOVA was conducted to determine the differences in working memory between the smartphone-addicted and non-addicted groups. The relationship between smartphone addiction and physical activity was analyzed using Pearson's chi-squared test.

**Results:**

The cognitive function-attention domain accuracy component showed a statistically significant difference between the groups, with a *p*-value of 0.05). The reaction time between smartphone-addicted and non-addicted children showed no statistically significant difference (*p* = 0.817). The relationship between smartphone addiction and physical activity was statistically significant (*p* < 0.001).

**Discussion:**

The interaction effects between physical activity and smartphone addiction on reaction times showed statistically insignificant (*p* = 0.25) differences, showing that physical activity's effect on reaction times did not depend on smartphone addiction levels. The non-addicted children had significantly higher physical activity levels than the addicted children, indicating that smartphone addiction reduced physical activity.

## Introduction

Due to the rapid expansion of the Internet and other technological breakthroughs, it is anticipated that the number of mobile phone users will continue to increase annually. Smartphones are considered the most prevalent electronic device among children. A study discovered that, compared to tablets and laptops, smartphones were the most commonly used gadgets among children, with a mean weekly usage of 28.5 h (Alobaid et al., 2018). This could be considered a strong indicator of children's rapid exposure to smartphone usage. Children currently rely on smartphones for many things, including attending online classes, communicating, playing games, shopping, and watching entertaining videos. Children also use smartphones instead of books for reading and studying (Nasution, 2021). Smartphones can be used to access educational resources, stay connected with friends and family, and learn new skills. This way of life significantly impacts children's day-to-day activities, and they become too attached to their smartphones, which might lead to addiction (Nasution, 2021).

Smartphones are characterized by rapid technological development and increasing prevalence due to their flexibility in function, portability, and purpose of use. There have been numerous unresolved queries concerning the effects of smartphone addiction on cognitive functions. Moreover, the conclusive evidence is still limited and conflicted, especially among children (Wilmer et al., 2017). Studies found that students exposed to smartphones or receiving more mobile phone calls or text messages showed shorter response times and were less accurate on working memory tasks (Abramson et al., 2009; Thomas et al., 2010). In contrast, Wasmuth et al. (2022). did not find a relation between general smartphone use (time/frequency) and inattention (Wasmuth et al., 2022). Neurophysiological studies report that heavy smartphone use is associated with attention, number processing, and right prefrontal cortex excitability impairments. However, there were no significant differences in working memory or inhibitory control (Hadar et al., 2017). While there is no conclusive evidence that smartphones harm a child's cognitive function, some studies have produced alarming results. Paulus et al. investigated the association between screen media activity behavior, brain structure, and cognitive function changes. They found a significant association between changes in the structural characteristics of the brain and time spent on screens, including smartphones. They also found that some activities related to screening media and brain structures are associated with worse cognitive performance, while others are associated with better cognitive performance. It suggests that screen media activity is not “good or bad for the brain” (Paulus et al., 2019). More research is required to determine whether or not the use of electronic devices impacts cognitive function, particularly by children.

Physical activity and smartphone addiction are regarded as two health-related independent variables, yet they are interconnected (Wu et al., 2017; Li et al., 2020). The prolonged use of smartphones can affect physical health by reducing participation in physical activities, which leads to a decrease in muscle mass and an increase in fat mass, both of which are associated with bad health consequences (Kim et al., 2015). A recent review of the association between smartphone addiction and participation in sports and physical activity among children and adolescents revealed that an increase in smartphone addiction decreases physical activity and sports performance (Azam et al., 2020). Although a few studies indicate no significant association between smartphone addiction and physical activities, this is not the case in general (Buctot et al., 2020). Numerous studies have shown that the prolonged use of smartphones is highly correlated with sedentary lifestyles and physical inactivity (Fennell et al., 2019; Xiang et al., 2020).

Physical activity can raise dopamine levels and receptor binding rates in the human body, which helps reduce addictive behaviors (Roberts et al., 2012). Fewer smartphone users would be extremely likely to experience better cognitive functions in their daily lives (Hadlington, 2015). According to a cross-sectional study, physical activity is directly connected with enhanced cognitive function (Hamer and Chida, 2008). A systematic review described that regular physical activity has the most protective effect against cognitive decline (Blondell et al., 2014). Therefore, this raises questions regarding the potential effects of smartphone addiction on cognitive function and physical activity in children. However, no systematic research has examined the effects of smartphone addiction on cognitive function and physical activity in Saudi Arabian children aged 12–14 years. This study aimed to assess the effect of smartphone addiction on cognitive function and physical activity among middle-school children. Another aim of the study was to compare the cognitive function and physical activity of addicted and non-addicted middle-school children.

## Materials and methods

### Study design and setting

A cross-sectional study with an analytic and descriptive structure was adopted to conduct this study. The research was conducted between December 2021 and February 2022 at eight public and private middle schools for boys and girls in the Eastern Province of Saudi Arabia (Dammam, Al Khobar, and Dhahran).

### Sample size

The sample size was calculated based on the Raosoft online calculator at (http://www.raosoft.com/samplesize.html). The confidence level was 95%, and the significance level was 5%, with an expected prevalence of 87% of mobile phone usage among children and adolescents in Saudi Arabia (DOCOMO GN, 2011). The recommended sample size is 173. With a 10% dropout rate, the estimated total sample required for the current study was 200 children.

### Ethics approval

Ethics approval was obtained from the Institute Review Board of the University of Imam Abdulrahman Bin Faisal (IRB-PCS-2021-03-369). Each participant signs a written informed consent form prior to participating in the study.

### Selection of schools and participants

The study samples were drawn from eight schools chosen randomly from a list of schools using a lottery method. Following contact with the selected schools to conduct the study, a random selection of children from these schools was also made using random number generator software (https://www.random.org/). All selected children were healthy school-going children of Saudi nationality and both sexes, with an age range of 12–14 years in the Gregorian calendar. Children with anemia, diabetes mellitus, hypertension, obesity, asthma, or seizures, a history of vision or hearing problems, anxiety, attention deficits, sleep disorders, a history of smoking, neuromuscular disorders, and physical disabilities and those who are unable to pay attention to the researcher's instructions or read and understand the questionnaires and testing procedures were excluded from the study. Based on their SAS-SV scores, the children were categorized into the smartphone-addicted and non-addicted groups.

### Procedure

The researcher went to the selected schools and explained the study process to the principals. The principals gave their written informed consent once they were informed about the research process and agreed to the data being collected at their schools. Afterward, children were randomly selected. All participants were recruited from intermediate-school grades 7, 8, and 9, with equal numbers of students from each grade to control confounding factors. Then, the research process was explained to them in detail. Once they agreed, they signed the consent form and participated in the study. Finally, the parents gave their consent through the WhatsApp application. With the help of the school principal, the researcher was provided with a copy of the student's medical history report, and all participants were assessed for eligibility. Out of 200 children, 196 met the criteria for inclusion. The researcher then started collecting their demographic information, such as their gender, age, level of education, height, weight, and body mass index (BMI). Smartphone-related information was collected, such as the duration of smartphone use per day and the number of years the children owned the smartphones. These details were recorded for each child on a separate data entry sheet. The height was measured (in meters) using a measuring tape, and the digital weighing scale was used to measure the weight (in kilograms). The body mass index (BMI) was calculated by dividing weight in kilograms by height in meters squared. After obtaining the demographic data, each child participated in three evaluations: the first evaluation was for assessing smartphone addiction levels, the second for evaluating cognitive function, and the third for measuring physical activity levels. The smartphone addiction and physical activity levels were assessed by self-assessment paper-based questionnaires using the Arabic versions of the Smartphone Addiction Scale-Short Version and the Physical Activity Questionnaire for older children, respectively. The working memory and selective attention domains of cognitive function were evaluated using a laptop screen's digital version of the memory automaticity and Flanker tasks, respectively. During the test procedure, the children were individually seated in a quiet room with the investigator in front of a laptop screen. The investigator explained the tasks to the children, and once they were ready and understood the task's procedure, they started the actual tasks.

**Figure 1:**
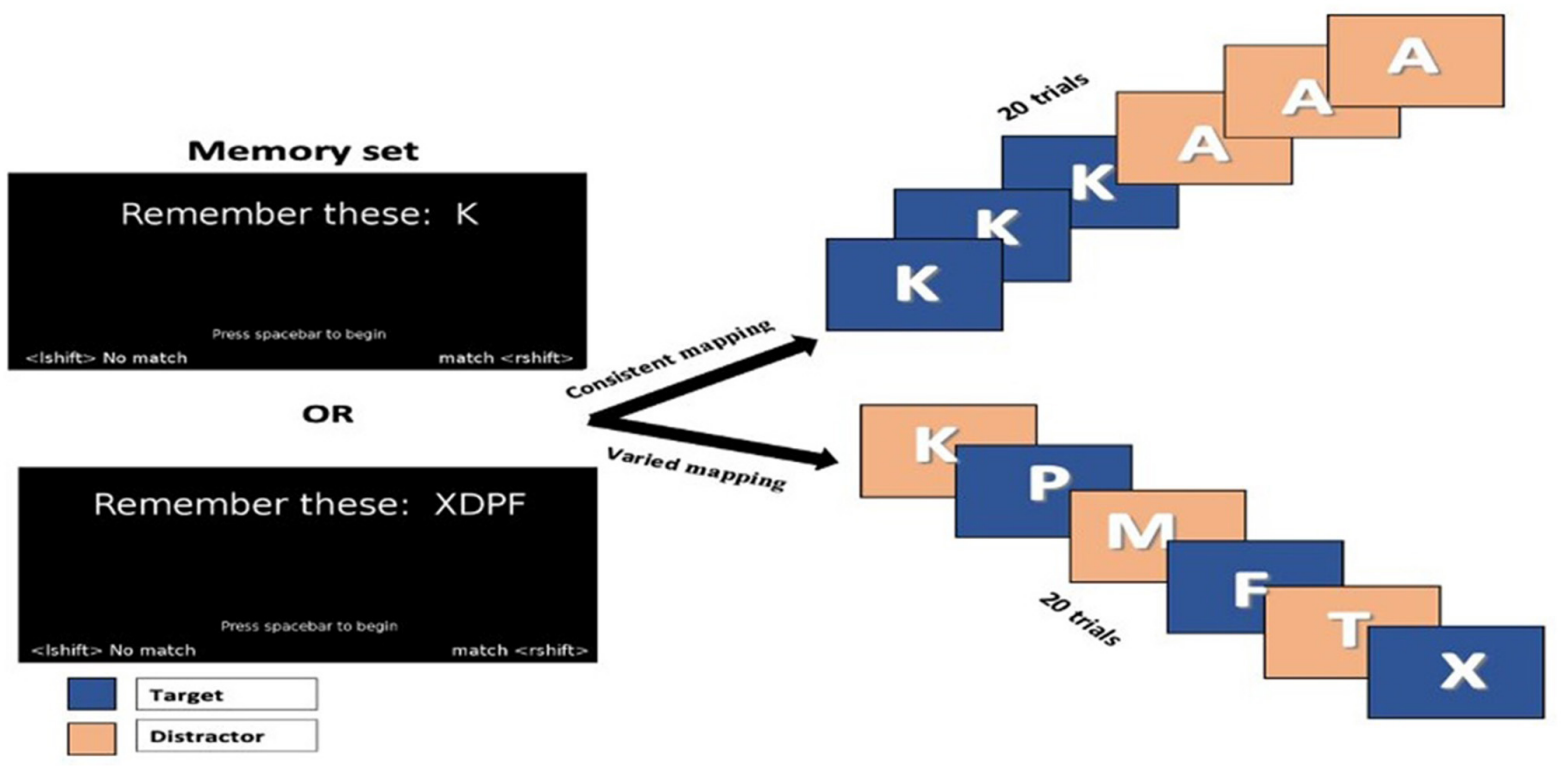
Illustration of the memory automaticity task for the assessment of working memory.

The memory automaticity task was used to assess working memory. This task requires remembering whether or not a letter is in a memory set and classifying it accordingly. A memory set is a set of learned alphabets designed to be recognized on a given trial. Letters that match any of the memorized items are called “targets,” while letters that do not match any of the memorized items are called “distractors.” Response times and accuracy are the dependent metrics. This task has two main components: consistent and varied mapping. Consistent mapping is used when the target and distractor items do not overlap across trials; they are mapped consistently; thus, this task is performed automatically and requires less focus and attention (Schneider and Shiffrin, 1977; Servant et al., 2018; Zhao et al., 2022). Varied mapping is used if the target and distractor items overlap each other. Hence, completing the test requires much more control, focus, and attention than consistent mapping (Schneider and Shiffrin, 1977; Zhao et al., 2022). First, the children were given a memory set of one to four alphabets at two different levels. They were asked to remember one to two alphabets in the first level, while the second level has three to four alphabets. This list of levels came randomly and could be consistent or varied (Figure 1). Once the participants memorized the alphabet in a memory set, they had to press the spacebar to begin; thus, the previous memory set would be deleted, and then, they were shown a series of alphabet inputs. They must decide whether each letter matches one of the alphabets in the memory set previously presented or not. When the letters do not match, they are considered distractors, and the participant responds using the left shift key. Alternatively, consider it a target when it matches and responds using the right shift key.

**Figure 2:**
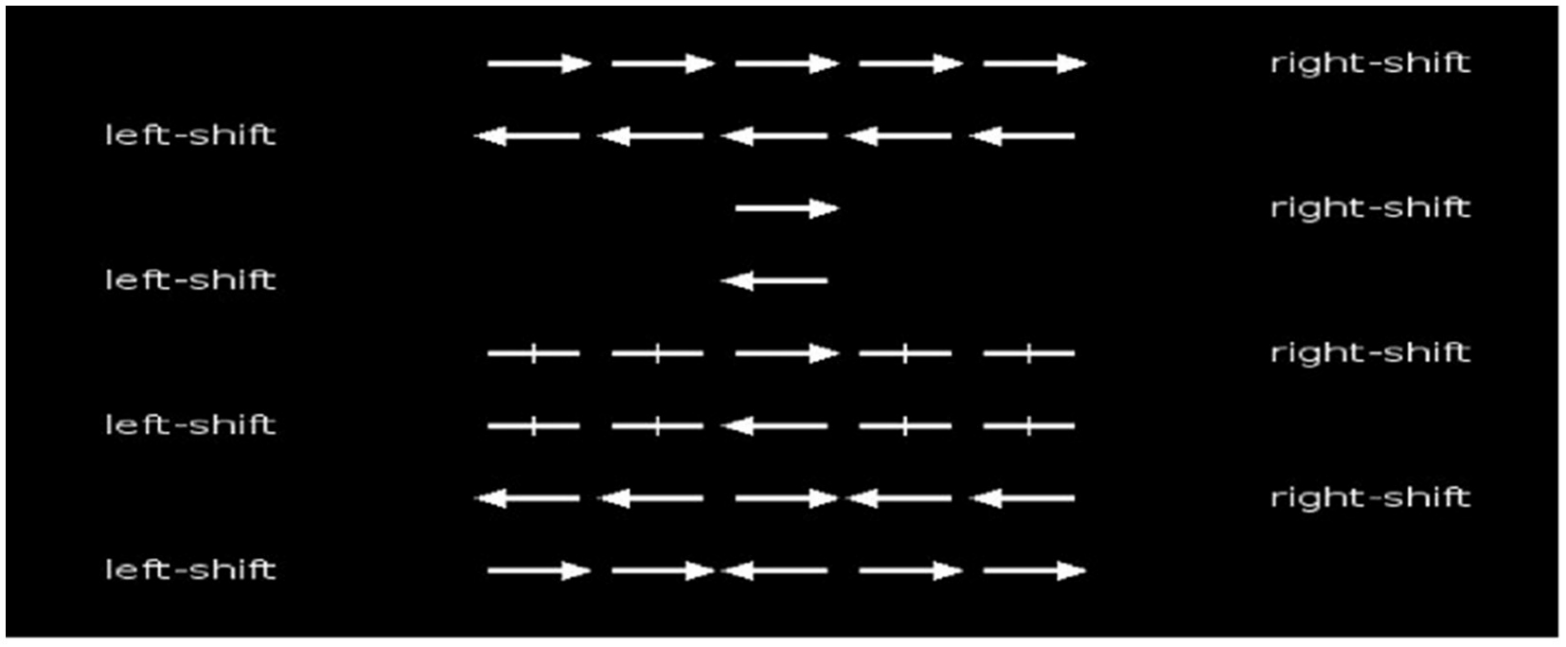
Illustration of the Flanker task for the assessment of selective attention.

The Flanker task assesses selective attention control by focusing on a stimulus while simultaneously inhibiting the detection of other stimuli (Servant et al., 2018). This task presented five randomly directed arrows in the screen's center. The participants were asked to determine the direction of the center arrow while ignoring the arrows in the periphery. Both hands' index and middle fingers were placed on the keyboard's right and left shift keys. The children were instructed to respond depending on the direction of the central arrow; if it points left, they should press the left shift key; if it points right, they should press the right shift key as fast as possible. The heads of the arrows surrounding the center arrow would either be in the same or the opposite direction, be absent, or only appear as lines (Figure 2). Before starting the main trials of the task, which had 96 trials, the children were allowed to practice for 10 trials. The overall mean reaction times in milliseconds and the mean accuracy were recorded for analysis. Data from the practice trials were excluded. The participants had approximately 3 min to complete the test. After obtaining all the required data, the children were categorized into the smartphone-addicted and non-addicted groups based on their SAS-SV scores. The scores of cognitive function and physical activity level were collected for further analysis.

### Outcome measurements

#### Smartphone addiction

Smartphone addiction levels were measured using the Arabic version of the Smartphone Addiction Scale-Short Version (SAS-SV) (Kwon et al., 2013a). It is a self-administered scale developed by Kwon et al. (2013a) and intended to assess smartphone addiction. The SAS-SV had 10 items assessed on a 6-point Likert scale, ranging from “one” for strongly disagree to “six” for strongly agree. The maximum score for the scale is 60, and the minimum score possible is 10 (Kwon et al., 2013a). The SAS-SV addresses five content areas: daily disturbances, tolerance, cyberspace-oriented relationships, overuse, and withdrawal (Haug et al., 2015). The original English scale version had excellent internal consistency, content, and concurrent validity (Kwon et al., 2013b). However, Sfendla et al. (2018) assessed the psychometric properties of the Arabic SAS-SV on the Moroccan sample and found excellent reliability (Sfendla et al., 2018). The Smartphone Addiction Scale-Short Version classified the users as addicts with scores of ≥31 for boys and ≥33 for girls or non-addicts with scores of < 31 for boys and < 33 for girls. This cutoff point is based on the original article that examined the validity and reliability of the SAS-SV questionnaire (Kwon et al., 2013a).

#### Cognitive function

The working memory and selective attention domains of cognitive function were assessed using the psychology experiment building language (PEBL) test battery. The PEBL is an open-source software system that is freely available for designing and conducting psychological experiments and is a versatile research tool for studying individual differences in neurocognitive performance (Piper et al., 2015). It has good reliability and validity (Piper et al., 2015). The battery can be freely downloaded from the website (http://pebl.sourceforge.net). However, under the PEBL battery, memory automaticity and Flanker tasks were employed to assess the working memory and selective attention domains, respectively.

#### Physical activity

The Arabic version of the physical activity questionnaire for older children was used to assess the children's physical activity. The PAQ-C is a self-administered scale designed to assess the children's physical activity in the last 7 days (Kowalski et al., 1997). The original scale has good reliability and validity and was invented to assess physical activity in children aged 8–14 (Benítez-Porres et al., 2016; Gobbi et al., 2016; Wang et al., 2016). In a recent study, the scale was translated into Arabic and proved excellent validity and reliability (Alharbi, 2019). The total number of items in the PAQ-C was nine. Each item on the scale was rated from 1 to 5, and the total score was the average of all the items (Kowalski et al., 1997). The total score ranges from 1 to 5, divided into a low physical activity score of ≤ 2.3, a moderate physical activity score of 2.4–3.7, and a high physical activity score of ≥ 3.8 (Alharbi, 2019).

### Statistics analysis

Data were transferred to a single Excel sheet, and all variables were analyzed using the Statistical Package for the Social Sciences software for Mac (IBM SPSS version: 28.0.1.0, New York, USA). The univariate analysis for the demographic characteristics and outcome measures was done using descriptive statistics. Descriptive statistics were reported as mean ± standard deviation for quantitative variables. Categorical variables were reported as frequencies and percentages. The normality of the variables' distribution was examined using the Shapiro–Wilk test. A one-way MANOVA was conducted to determine the differences in working memory between the smartphone-addicted and non-addicted groups. To illustrate smartphone addiction's effect on selective attention, the reaction times and accuracy means were compared between the two groups using an independent sample *t*-test. The relationship between smartphone addiction and physical activity was analyzed using the Pearson chi-squared test. The effect size was determined using Cohen's formula, which represents the average effect size as also *d* = 0.4, with 0.2, 0.4, and 0.6 considered small, medium, and large effects, respectively. The interaction effects between physical activity and smartphone addiction on reaction times were analyzed using a 2 × 3 factorial design. The criterion for statistical significance was set at a *p*-value of ≤ 0.05.

## Results

Based on their smartphone addiction scale-short version scores, the children were categorized into two groups: smartphone-addicted and non-addicted. Moreover, approximately half (49.5%) of the children used their phones for more than 5 h a day, and approximately two-thirds used mobile phones for 2–4 years (66.3%). The mean ± (SD) of age, height, weight, and body mass index of the included participants was 12.99 ± 0.81, 153.86 ± 6.50, 48.07 ± 7.31, and 20.22 ± 2.08, respectively (Table 1).

**Table 1:** Demographic characteristics of total participants and differences between the smartphone-addicted and non-addicted children.

**Mean, standard deviation (SD) and frequency distribution of children' characteristics**	**Addicted (*n =* 100)**	**Non-addicted (*n =* 96)**	**Total (*n =* 196)**
**Mean (SD)**			
Age in years	13.11 (0.82)	12.88 (0.78)	12.99 ± 0.81
Height in meters	154.56 (6.40)	153.13 (6.55)	153.86 ± 6.50
Weight in kilograms	48.80 (6.41)	47.31 (8.10)	48.07 ± 7.31
Body mass index kg/(m)^2^	20.38 (1.93)	20.05 (2.22)	20.22 ± 2.08
**Frequency (Percent)**			
Gender			
Male	44 (44 %)	54 (56.3 %)	98 (50 %)
Female	56 (56 %)	42 (43.8 %)	98 (50 %)
**Education level**			
Middle-school grade 1	29 (29 %)	37 (38.5 %)	66 (33.7 %)
Middle-school grade 2	30 (30 %)	34 (35.4 %)	64 (32.7 %)
Middle-school grade 3	41 (41 %)	25 (26 %)	66 (33.7 %)
**Daily smartphone usage time**			
< 1 h per day	0 (0.0 %)	22 (22.9 %)	22 (11.2 %)
< 4 h per day	9 (9 %)	68 (70.8 %)	77 (39.3 %)
More than 5 h per day	91 (91 %)	6 (6.3 %)	97 (49.5 %)
**Years of smartphone ownership**			
1 year and less	14 (14 %)	28 (29.2 %)	42 (21.4 %)
2–4 years	69 (69 %)	61 (63.5 %)	130 (66.3 %)
More than 5 years	17 (17 %)	7 (7.3 %)	24 (12.2 %)

**Figure 3:**
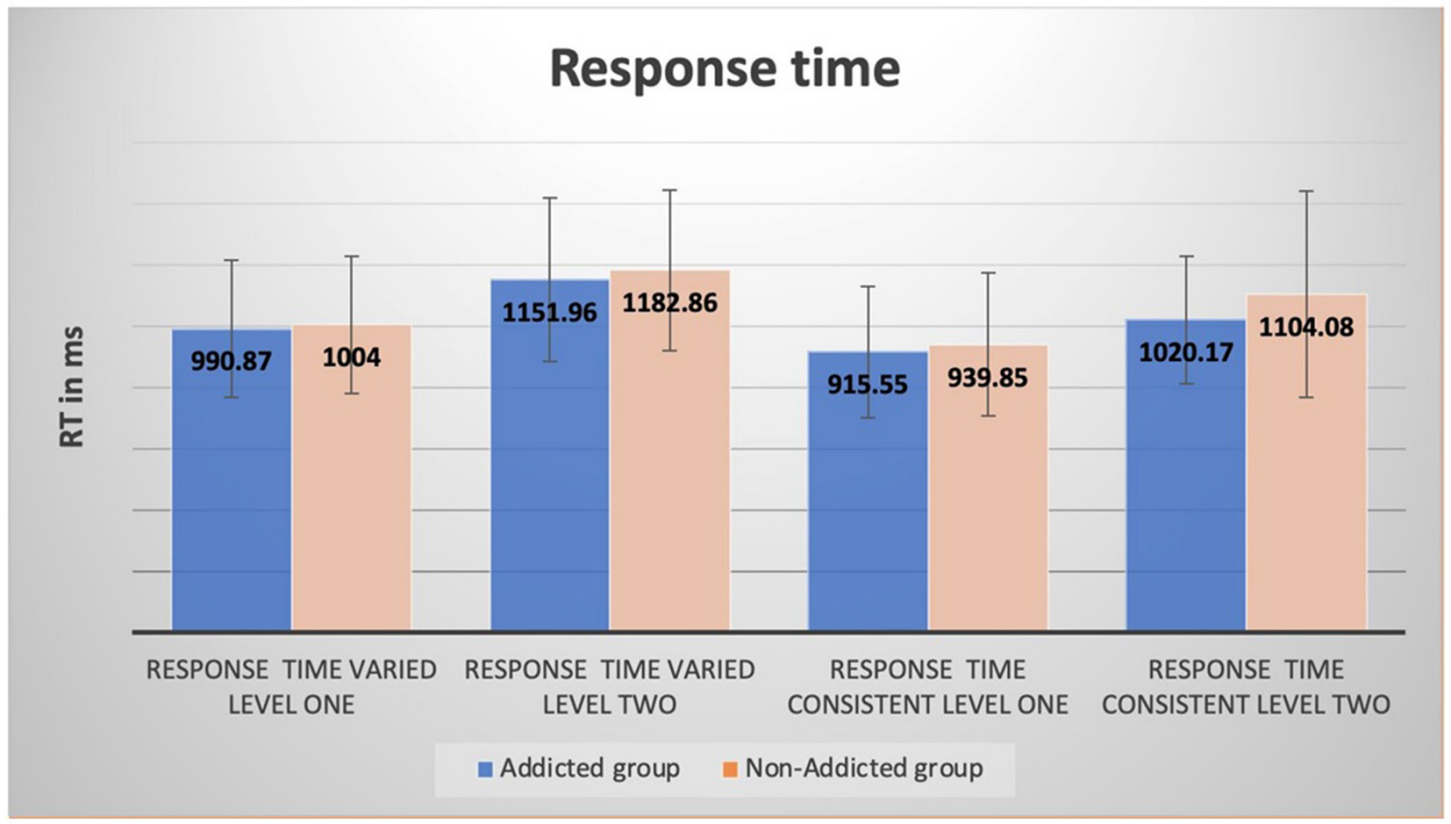
Differences between smartphone-addicted and non-addicted children in response time for varied and consistent components at levels 1 and 2.

**Figure 4:**
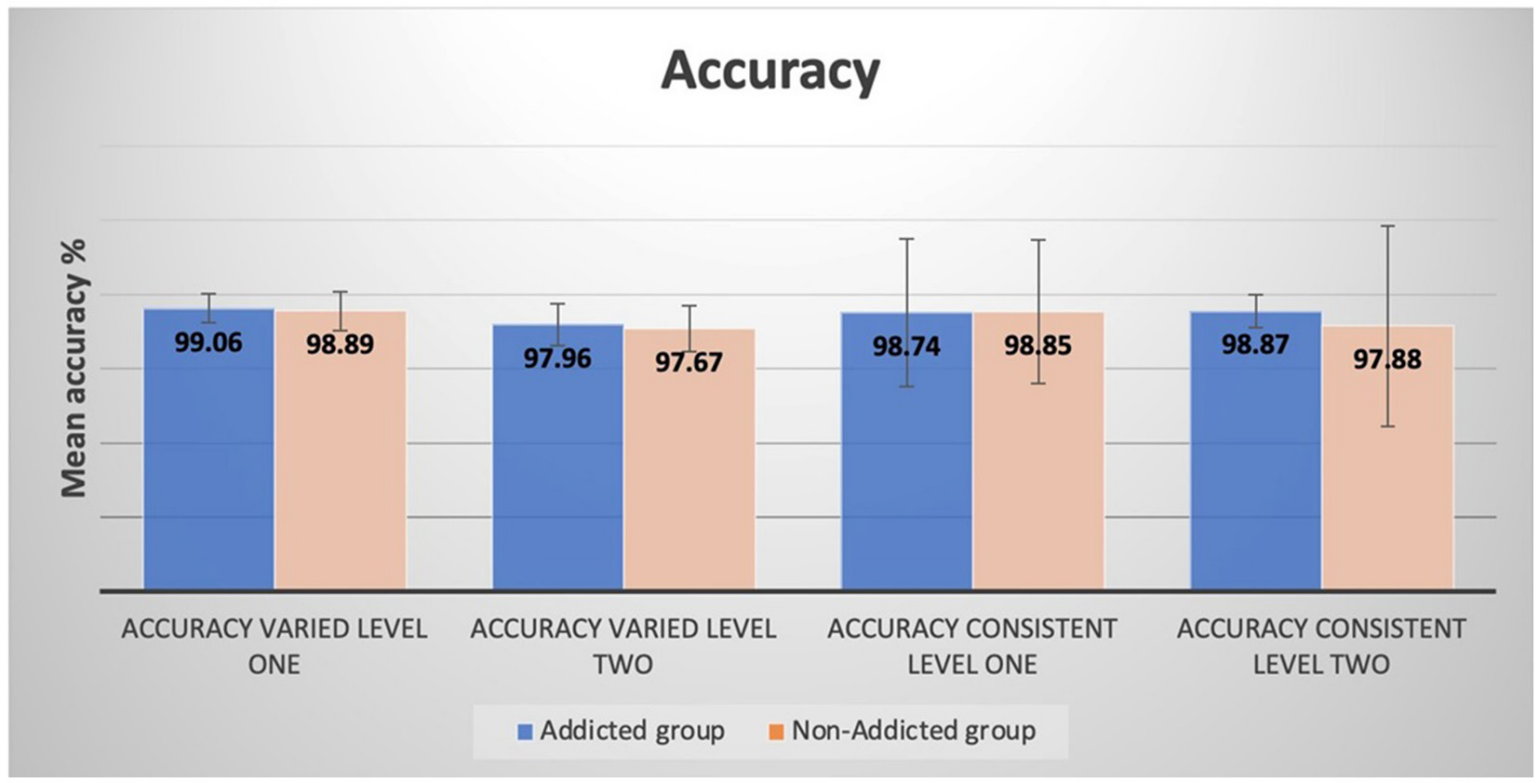
Differences in accuracy between smartphone-addicted and non-addicted children for varied and consistent components at levels 1 and 2.

The difference between the addicted and non-addicted children in response times for both varied, and consistent components was not statistically significant; *F*_(4, 191)_ = 1.154, *p* = 0.333; Wilks' Λ = 0.976; partial η^2^ = 0.024. Furthermore, the difference between the addicted and non-addicted children on accuracy for both varied and consistent components was not statistically significant; *F*_(4, 191)_ = 0.968, *p* = 0.426; Wilks' Λ = 0.980; partial η^2^ = 0.020. The mean response times and accuracy differences between smartphone-addicted and non-addicted children showed that the addicted children had shorter response times and were more accurate than the non-addicted children (Figures 3, 4).

Table 2 shows that the independent sample *t*-test has shown no statistically significant difference in reaction times between smartphone-addicted and non-addicted children (*t* = 0.464, *p* = 0.817). However, the accuracy component showed a statistically significant difference between the groups (*t* = 2.617, *p* = 0.005). The mean accuracy shows that smartphone-addicted children have a higher accuracy rate than non-addicted children. The small effect (*d* = 0.066) was shown between smartphone-addicted and non-addicted children for reaction times, whereas a medium effect (*d* = 0.374) size was shown for accuracy.

**Table 2:** Reaction times and accuracy differences between smartphone-addicted and non-addicted children.

	**Children's groups**	**Mean**	**Standard deviation**	**95% CI**		***t***	***p*-value**	**Effect size (d)**
				**Lower**	**Upper**			
**Reaction times (RT)**	Addicted	465.73	55.45	−12.138	19.611	0.464	0.817	0.066
	Non-Addicted	461.99	57.23	−12.149	19.622			
**Accuracy %**	Addicted	91.88	6.72	0.744	5.300	2.617	0.005^*^	0.374
	Non-Addicted	88.86	9.29	0.727	5.316			

^*^Significant at 0.05 level.

Two-way cross-tabulation shows that smartphone-addicted children had lower levels of physical activity. In contrast, non-addicted children had moderate-to-high levels of physical activity. In addition, the Pearson chi-square test showed that this relationship was statistically significant (X^2^ = 84.60, *p* < 0.001). A large effect size (phi = 0.657) can be observed between addicted and non-addicted children for physical activity levels (Table 3).

**Table 3:** Relationship between smartphone addiction and physical activity.

		**Physical activity levels (number)**			**Pearson chi-square (X^2^)**	***p*-value**	**Effect size (phi)**
		**Low**	**Moderate**	**High**			
**Children's groups**	**Addicted (*****n** **=*** **100)**	61	39	0	84.60	< 0.001^*^	0.657
	**Non-addicted (*****n** **=*** **96)**	10	38	48			

^*^Significant at 0.05 level.

Table 4 shows no statistically significant (*p* = 0.250) differences among smartphone-addicted and non-addicted children for low and moderate-to-high physical activity subgroups. Partial eta squared (η^2^ = 0.007) showed very little effect between addicted and non-addicted children for the level of physical activity.

**Table 4:** Interaction effects between physical activity and smartphone addiction on reaction times.

**Children's groups**	**Physical activity levels**	**Mean**	**SD**	**95% CI**		***F***	***p*-value**	**Partial eta squared**
				**Lower**	**Upper**			
**Addicted**	**Low**	457.41	55.025	443.211	471.607	1.330	0.250	0.007
	**Moderate**	478.74	54.281	460.988	496.501			
**Non-addicted**	**Low**	469.23	52.791	434.158	504.292			
	**Moderate**	463.96	51.576	445.973	481.951			
	**High**	458.93	63.001	442.923	474.934			

## Discussion

The current study aimed to investigate the effects of smartphone addiction on cognitive function and physical activity in middle-school children. According to the present study, there are no significant differences in working memory or reaction times for selective attention tasks between smartphone-addicted and non-addicted children. A significant difference was observed only in the accuracy component of the selective attention task, indicating that the smartphone-addicted children were more accurate than the non-addicted children. Concerning the differences in physical activity between smartphone-addicted and non-addicted children, the present study's results indicate that non-addicted children were significantly more active than smartphone-addicted children. Concerning the interaction effects between smartphone addiction and physical activity, the results have shown no significant interaction effects between physical activity and smartphone addiction on reaction times.

In the present study, mean response time and accuracy values indicated that the smartphone-addicted children performed the working memory task slightly better than the non-addicted children at each level. The smartphone-addicted children had shorter mean response times and were more accurate than the non-addicted children for varied and consistent mapping components. These findings suggest that smartphone-addicted children might remember the selected target faster and more accurately than non-addicted children might, which contradicts the current study's hypothesis. Most of the children in this study had been repetitively using their smartphones for more than 5 h per day for 2–4 years. Therefore, considering that smartphone overuse could enhance the user's sensory-motor coordination, decrease their response time, and increase their accuracy (Grewal and Sahni, 2019; Jordan and Dhamala, 2022). Moreover, the current study's working memory assessment depends on a computer-based task. As a result, smartphone-addicted children could complete tasks better and more efficiently than non-addicted children as they are more familiar with using such devices than non-addicted children.

Furthermore, working memory performance may improve with practice because of the brain's plasticity (Jak, 2012; Choudhury and McKinney, 2013). Imren and Tekman found that media multitasking improves working memory but inhibits the ability to sustain attention. They hypothesized that these results might have occurred because multitasking requires working memory practice, and media multitasking involves switching between devices or their functions (Imren and Tekman, 2019). Therefore, working memory performance can increase with practice, improving cognitive function (Jak, 2012; Choudhury and McKinney, 2013; Loh and Kanai, 2016). In addition, Tanaka et al. (2013) indicated that smartphone addicts had greater gray matter volume in the posterior parietal cortex, which was associated with better visual working memory performance (Tanaka et al., 2013). However, statistically, there are no significant differences in working memory between smartphone-addicted and non-addicted children. The lack of statistically significant differences in the memory (accuracy) task may be attributed to the limited differences between the addicted and non-addicted children with regard to cognitive functions, making it difficult to draw statistically significant differences. Studies have demonstrated that childhood and adolescence are characterized by the continuous development and maturation of various prefrontal cortex-mediated behaviors, including planning, attentional control, working memory, inhibitory control, and decision-making (Hooper et al., 2004; Conklin et al., 2007; Luciana et al., 2009).

The attention domain of cognitive function assessed by the Flanker task is based on reaction times in milliseconds and accuracy. Regarding reaction times, the current study showed no statistically significant difference between smartphone-addicted and non-addicted children. In a recent neurophysiological study, researchers aimed to determine whether excessive smartphone use is accompanied by measurable neural, cognitive, and behavioral changes. They conducted a longitudinal experiment to identify smartphone use's effects on the participants' cognitive functions and to observe the differences between heavy smartphone users and non-users. They found that heavy smartphone users were experiencing hyperactivity and increased impulsivity. Moreover, heavy smartphone users had reduced early transcranial magnetic stimulation-evoked potentials induced by transcranial magnetic stimulation on the right side of the prefrontal cortex compared to non-smartphone users, which were associated with self-reported inattention problems. However, the researchers did not observe significant differences between the groups' memory domains (Hadar et al., 2017). Consistent with the study mentioned above, the current study found no significant differences in the memory domain of cognitive function between smartphone-addicted and non-addicted children. However, regarding the attention function, the current study's findings contradict the previous study's results. The current study's results showed no statistically significant difference between smartphone-addicted and non-addicted children in the reaction time of the attention domain. In contrast, the prior study found that heavy smartphone use was significantly associated with inattention problems. The differences in results could be attributed to the methodological differences in how the studies were conducted.

The study mentioned above used the Conners Adult ADHD Rating Scales (CAARS) questionnaire to assess inattention, whereas the present study used a computer-based test. Paper-based questionnaire assessments are different from computer-based tests. In paper-based tests, all the questions are in front of the participant at once, allowing them to move between questions as they wish, which may increase the chance of bias and error. In computer-based tests, by contrast, questions are presented one after the other with limited time to complete the task; therefore, participants have no opportunity to return to previously posed questions, which may help provide more accurate results of the attention function. However, we currently lack evidence to support this intuitive interpretation, so we cannot completely exclude it.

The above-described study's sample included only adults, whereas the current study's participants were children. The developmental stage of childhood is characterized by ongoing neurological growth, which distinguishes children from adults (Larsen and Luna, 2018). There is substantial evidence to indicate that children influence attentional performance. For instance, the capacity to sustain attention, inhibit inappropriate responses, and shift attentional focus improves throughout childhood (Halperin et al., 1991; Greenberg and Waldman, 1993). Therefore, it is difficult to definitively judge smartphone addiction's effect on children's attention functions. However, the difference in the effects of smartphone addiction on attention function between adults and children indicates the possibility of adverse long-term effects. These results sparked further curiosity of the current study's team to move research forward, conduct a longitudinal study, and include a wide range of age groups to uncover more results.

The current study's findings have shown no statistically significant difference in reaction times between smartphone-addicted and non-addicted children. However, smartphone-addicted children had a significantly higher accuracy rate than non-addicted children in the attention task. One possible explanation for the smartphone-addicted children's high accuracy rate could be the experience and skills acquired by these children from using smartphones. Smartphone-addicted children have been using the smartphone, repetitively, for an extended period, which could have enhanced their neuronal circuits, thus increasing their ability to filter irrelevant information. In a series of studies by Dye et al. (2009), and Bavelier et al. (2012) increased media multitasking was associated with better attention control; they found that smartphone-addicted participants were better at inhibiting irrelevant information than other groups (Dye et al., 2009; Bavelier et al., 2012). In addition, a recent study found that smartphone-addicted participants were more attentive than their counterparts (Alsaad et al., 2022).

Regarding smartphone addiction's effect on physical activity, the current study confirmed the link between smartphone addiction and low physical activity. The findings showed that smartphone-addicted children were less physically active. In contrast, non-addicted children are likelier to have moderate-to-high physical activity levels. Furthermore, this association was statistically significant. In agreement with the present study's results, Azam et al. (2020) conducted a systematic review to emphasize the links between smartphone addiction, sports participation, and physical activity. Their review included eight global studies, all of which had been conducted on children and adolescents. All the studies included in their review showed similar results, demonstrating that an increase in smartphone use leads to decreases in physical activity and sports performance among children and adolescents (Azam et al., 2020). Similarly, Wang et al. (2016) revealed that smartphone addiction decreases physical fitness among university students (Li et al., 2022).

The present study's secondary objective was to identify the interaction effects between physical activity and smartphone addiction on reaction times. It could be considered that the effects of physical activity on reaction times depend on the levels of smartphone addiction. Therefore, smartphone-addicted and non-addicted children were divided into low and moderate-to-high physical activity subgroups based on the physical activity questionnaire. However, there were no significant interaction effects between physical activity and smartphone addiction on reaction times. These findings indicate that the effect of physical activity on reaction times did not depend on whether the children were addicted or non-addicted to smartphones. These results could be attributed to the fact that reaction times depend on many other factors, including gender (Naglieri and Rojahn, 2001), sleep quality (Paavonen et al., 2010), children's birth order, residence, breakfast intake, and the mother's smoking history (Almomani et al., 2014). However, the current study did not investigate such factors' effects on reaction times. Notably, studies have yet to examine the interaction effects between physical activity and smartphone addiction on reaction times.

This study also has several limitations, which indicate possibilities for further investigation. First, the cross-sectional study design prevents any cause–effect relationship. Further longitudinal investigation is required to determine the directionality of the investigated correlations. Second, the sample was a specific age group, and all participants were recruited only from schools in the eastern region, which may affect the likelihood of generalizability. Third, there was no intervention used to determine the cause-and-effect assumptions. Fourth, self-reported questionnaires were used to determine the level of physical activity and smartphone addiction behavior that may lead to biases. Fifth, the study investigated smartphone addiction in only two cognitive domains: working memory and selective attention. Future researchers can investigate the impact of smartphone addiction on other neurocognitive domains, such as problem-solving and planning, helping to highlight the other cognitive domains that could be affected by smartphone addiction. Finally, smartphone addiction is complicated and multidimensional. Thus, examining the varied activities, contents, and patterns of smartphone use in future research would be beneficial.

## Conclusion

The present study demonstrated that smartphone-addicted children were significantly more accurate than non-addicted children. Non-addicted children had significantly higher physical activity levels than addicted children. Smartphone-addicted children have shorter response times and are more accurate than non-addicted children in working memory tasks for varied and consistent mapping. In addition, the current study showed no significant interaction effects between physical activity and smartphone addiction on reaction times, indicating that the effect of physical activity on reaction times did not depend on smartphone addiction levels. Further studies are required to corroborate findings and aid in developing preventative and intervention measures.

## Data availability statement

The original contributions presented in the study are included in the article/supplementary material, further inquiries can be directed to the corresponding author.

## Ethics statement

The studies involving human participants were reviewed and approved by the Imam Abdulrahman Bin Faisal University, Dammam. Written informed consent to participate in this study was provided by the participants' legal guardian/next of kin.

## Author contributions

AA-A, SA, and TA: conceptualization. AA-A: data curation, investigation, and writing—original draft. SB: formal analysis and software. AA-A and TA: methodology. SB and MA: resources and writing—reviewing and editing. SA, MA, and TA: supervision. SA and TA: validation. SA and MA: visualization. All authors contributed to the article and approved the submitted version.
